# Yanshu spraying agent, a traditional Chinese medicine, relieves chronic pharyngitis in animals by anti-inflammatory and antibacterial effects

**DOI:** 10.3892/etm.2014.1524

**Published:** 2014-02-06

**Authors:** CHENGWEN LU, YANQIN SONG, JIANQIAO ZHANG, YUAN DU, TIAN WANG, YUNLI XUE, FENGHUA FU, LEIMING ZHANG

**Affiliations:** 1 Department of Pharmacology, School of Pharmacy, Yantai University, Yantai, Shandong 264005, P.R. China; 2 Shandong Luye Pharmaceutical Company, Ltd., Yantai, Shandong 264003, P.R. China

**Keywords:** chronic pharyngitis, Yanshu spraying agent, anti-inflammatory effects, antibacterial effects

## Abstract

Chronic pharyngitis is chronic inflammation that is often caused by repeated occurrences of acute pharyngitis or upper respiratory tract infections, including *Streptococcus* and *Staphylococcus*. The present study aimed to investigate the effects of Yanshu spraying agent (Yanshu) in relieving chronic pharyngitis, as well as the possible underlying mechanisms. The results revealed that Yanshu inhibited chronic inflammation in ammonia-induced chronic pharyngitis in rabbits and cotton pellet-induced granuloma tissue formation in rats. Yanshu also demonstrated antibacterial effects on *Streptococcus* and *Staphylococcus in vitro*. Yanshu did not exhibit any effects on the immune system, including the spleen and thymus indexes, immunocyte count and monocyte-macrophage function, when compared with the effects of dexamethasone. Therefore, the results of the present study indicate that Yanshu may relieve chronic pharyngitis via its anti-inflammatory and antibacterial activities.

## Introduction

Chronic pharyngitis, a chronic inflammation of the pharyngeal mucous membrane and submucous lymphoid tissues, is often caused by unsatisfactory treatment of acute pharyngitis. The disease may also occur in response to chronic inflammation caused by alcohol abuse, overuse of the voice and cigarettes. Clinically, chronic pharyngitis manifests itself as itching, dryness, soreness of the throat, coughing and the feeling of a foreign body or obstruction in the throat (1). Chronic pharyngitis may have an infectious or non-infectious etiology. In bacterial pharyngitis, group A β-hemolytic *Streptococcus* and *Staphylococcus aureus* are most frequently (5–36%) isolated (2,3). Chronic pharyngitis may be caused by immune dysfunction of the body, as the immune system is insufficient in suppressing persistent microbial infection caused by bacteria, including β-hemolytic *Streptococcus* and *Staphylococcus aureus* (4).

Treatments for chronic pharyngitis vary depending on the underlying cause. Immediate treatment includes anti-inflammatory medications to reduce the swelling in the throat and improve the comfort of the patient, as well as antibiotic or antiviral medications that target infectious organisms in the throat. Yanshu spraying agent (Yanshu), a potential therapeutic agent for pharyngitis in clinical practice, consists of 11 herbs, including Radix Sophorae Tonkinensis*,* Calyx seu Fructus Physalis Francheti and Radix Scutellariae Baicalensis. A previous study demonstrated that Yanshu exhibits potent anti-inflammatory activity on the xylene-induced ear and carrageenan-induced paw edema models, which may be mediated by inhibiting the expression of cyclooxygenase-2 (5). However, the pharmacological effects of Yanshu on chronic pharyngitis have not yet been demonstrated. Thus, the aim of the present study was to investigate the effects of Yanshu on chronic pharyngitis in animals and the anti-inflammatory and antibacterial mechanisms.

## Materials and methods

### Drugs and chemicals

Yanshu was provided by Shandong Luye Pharmaceutical Co, Ltd. (20120525; Yantai, China). Yanshu is composed of 11 plant materials, including Radix Sophorae Tonkinensis, Calyx seu Fructus Physalis Francheti, Periostracum Cryptotympanae, Radix Rehmanniae, Fructus Arctii, Radix Scutellariae Baicalensis, Radix Paeoniae Rubra, Semen Qroxyli, Semen Sterculiae Lychnophorae, Cortex Moutan Radicis and Herba Menthae Haplocalycis, at a ratio of 1.7:1.7:1.7:2.4:1.7:1.7:2:1.7:1.7:1.7:1.7, respectively (dry weight). All plant materials were selected according to the pharmacopoeia of the People’s Republic of China (2010) (6). Yanshu (2 kg) was prepared as a mixture of the aforementioned components and extracted with 24 and then 20 liters distilled water at 100°C for 1.5 h, respectively. After filtering the liquid, the extract was concentrated with a rotary vacuum evaporator to reach a concentration of 2.0 g/ml which was stored at 4°C.

Diclofenac diethylamine emulsion (Votalin) was purchased from Beijing Novartis Pharma Co., Ltd (X0587; Beijing, China). Penicillin round paper (diameter, 6 mm; content, 10 μg) was purchased from Hangzhou Tianhe Microorganism Reagent Co., Ltd (111209; Hangzhou, China) and dexamethasone was purchased from Shandong Lukang Cisen Pharmaceutical Co., Ltd (120229203; Jining, China).

### Animals

Male Swiss mice (weight, 18–22 g) and male Wistar rats (weight, 160–200 g) were purchased from Beijing Vital River Laboratories Animals Technology Co., Ltd (Beijing, China). New Zealand white rabbits (weight, 2.5–3 kg) were purchased from the Experimental Animal Center of Shandong Luye Pharmaceutical Co., Ltd. (Yantai, China). All experimental procedures in the study were performed in accordance with the guidelines for the Care and Use of Laboratory Animals from the National Institute of Health and were approved by the Ethics Committee of Yantai University (Yantai, China). All the animals were housed in diurnal lighting conditions (12:12 h) and provided with access to food and water *ad libitum*.

### Chronic pharyngitis animal model

The chronic pharyngitis animal model was established as previously described (7), but with minor modifications. Briefly, rabbits were sprayed with 2.5% ammonia water into the pharynx mucosal twice per day (600 μl total) for 15 consecutive days. On day 8, 0.5 ml oil of turpentine was injected into the pharynx mucosal of the rabbits. The rabbits were randomly divided into the control, model, watermelon frost (reference drug used in acute and chronic pharyngitis) and Yanshu low-, medium- and high-dose groups (n=5 per group). Each treatment group received the respective treatment of vehicle, 200 mg watermelon frost or 300 μl Yanshu (0.5, 1 or 2 g/ml) sprayed into the pharynx mucosal four times a day for 14 consecutive days. After 24 h following the last administration, animals were anesthetized and pharyngeal tissue was removed and fixed in 4% formalin. The rabbits were anesthetized with urethane (1 g/kg). Tissues were sliced for hematoxylin and eosin staining to observe pathological differences between the groups under a light microscope (Olympus Co., Ltd., Beijing, China).

### Cotton pellet-induced granuloma tissue formation test

The cotton pellet-induced granuloma tissue formation animal model was established as previously described (8), but with minor modifications. Briefly, rats were anesthetized with 350 mg/kg chloral hydrate and two sterilized cotton pellets, that weighed 20 mg each, were implanted subcutaneously on each side of the nape through a small ventral incision in the nape of the rats. Following implantation of the cotton pellets, each rat treatment group (n=10 per group) received topical treatment of vehicle, 100 mg Votalin or 300 μl Yanshu at 0.5, 1 or 2 g/ml, four times a day for seven consecutive days. Eight days following implantation of the cotton pellets, the animals were anesthetized with chloral hydrate. Each implanted cotton pellet was removed with the surrounding fibrovascular tissue and dried at 60°C for 12 h. The dry weight was then measured and the net granuloma weight was calculated by minusing the original pellet weight from the dry pellet weight. The granuloma inhibition rate was calculated as follows: Granuloma inhibition rate (%) = 1 - (drug group average granuloma net weight)/(control group average granuloma net weight) × 100.

### Antibacterial test in vitro

Antibacterial tests were performed using the cylinder-plate method (9). A tested 200 μl bacteria suspension (β-hemolytic *Streptococcus* and *Staphylococcus aureus*; concentration, 1×10^7^/ml) was daubed by SS-Spreader (Egoan Technology Co., Ltd., Beijing, China) on culture medium (*Staphylococcus* was cultured using nutrient agar plate and β-hemolytic *Streptococcus* using blood plate) under aseptic conditions. The ready plates were dried at room temperature for 15 min and the cylinder-plates (6×8×10 mm) were placed on the medium. Normal saline (200 μl) or varied concentrations of Yanshu (2, 1, 0.5, 0.25, 0.125, 0.0625 and 0.03125 g/ml; 200 μl) were added to the cylinder, respectively. Plates were then stored at 4°C for 2 h and cultured at 37°C for 24 h. The diameters of the effective inhibitory zone of Yanshu were measured.

### Spleen index, thymus index and immunocyte count

In total, 50 mice were randomly divided into vehicle, dexamethasone (5 mg/kg body weight per day) and three Yanshu groups (10, 20 and 40 g/kg body weight per day; n=10 per group). Mice received oral administration of the corresponding drugs once per day for seven consecutive days. After 24 h following the last administration, blood samples were collected for leukocyte and lymphocyte counts. Mice were then sacrificed and the weight of the spleen and thymus were measured. The index was calculated as follows: Organ index = organ weight (mg)/body weight (g).

### Carbon particle clearance test

Phagocytosis rates of monocytes and macrophages were estimated by calculating the speed of carbon particle clearance. The carbon particle clearance test was performed as previously described (1), but with minor modifications. Briefly, 50 mice were randomly divided into a vehicle, dexamethasone (5 mg/kg body weight per day) and three Yanshu groups (10, 20 and 40 g/kg body weight per day; n=10 per group). Mice received oral administration of the corresponding drugs once a day for seven consecutive days. After 2 h following the last administration, Indian ink (0.1 ml/10 g; Beijing Xizhong Chemical Co., Ltd., Beijing, China) was injected intravenously and 20 μl blood samples were collected following 3 min and 13 min. The blood samples were hemolyzed in 1 ml Na_2_CO_3_ (0.1%) and the concentration of carbon particles was determined by measuring the optical density (OD) at 600 nm. The clearance index (CI) and phagocytic index (PI) were calculated as follows: CI (K) = [logOD_1_ (control) - logOD_2_ (treated)]/t_1_–t_2_; and PI = body weight/(liver weight + spleen weight) ×^3^√k.

### Statistical analysis

Data are expressed as the mean ± SD. Data were analyzed using one-way analysis of variance with the Bonferroni post hoc test used for multiple t-tests. P<0.05 was considered to indicate a statistically significant difference.

## Results

### Effect of Yanshu on chronic pharyngitis in rabbits

**Figure 1:**
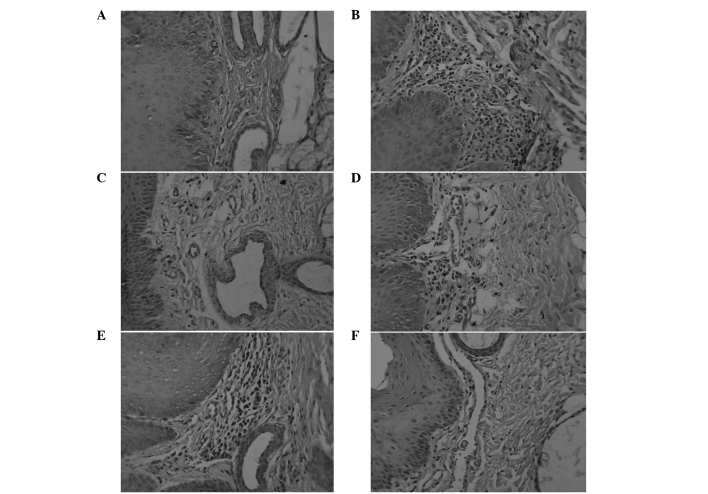
Histopathology of the pharyngeal tissue induced by 2.5% ammonia in rabbits in (A) control, (B) model, (C) watermelon frost, (D) Yanshu low-dose, (E) Yanshu medium-dose and (F) Yanshu high-dose groups.

Under a light microscope, a large number of inflammatory cells were observed in the pharyngeal tissue following administration of 2.5% ammonia and the number of lymphocytes increased significantly. Compared with the model group, the watermelon frost (reference drug) and Yanshu high-dose treatment groups showed a significantly reduced number of inflammatory cells (Fig. 1).

### Effect of Yanshu on chronic granulomatous inflammation in rats

**Figure 2:**
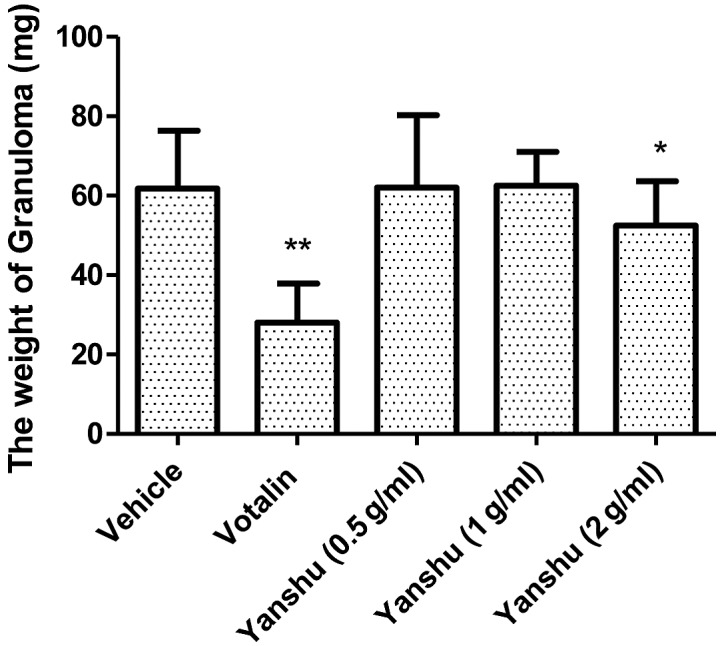
Effects of Yanshu on chronic granuloma inflammation in rats, as measured by granuloma weight. Results are expressed as the mean ± SD (n=10). ^*^P<0.05 and ^**^P<0.01, vs. vehicle group.

When treated with Votalin or high-dose Yanshu, the granuloma weight decreased significantly (P<0.01 and P<0.05, respectively) as compared with the model group (Fig. 2).

### Antibacterial activity of Yanshu in vitro

Yanshu demonstrated antibacterial activity against *Staphylococcus aureus* (≥0.125 g/ml) and β-hemolytic *Streptococci* (≥1 g/ml). The results are shown in Table I.

### Effect of Yanshu on immune organs and immune cells

**Figure 3:**
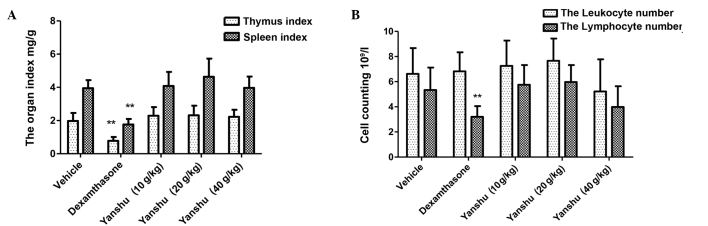
Effects of Yanshu on the (A) thymus and spleen indexes and (B) number of leukocytes and lymphocytes in mice. Results are expressed as the mean ± SD (n=10). ^**^P<0.01, vs. vehicle group.

Compared with the vehicle group, the thymus and spleen indexes in the Yanshu-treated mice did not change significantly. However, the dexamethasone-treated mice exhibited a significant decrease in thymus and spleen indexes (P<0.01; Fig. 3A). There were no marked changes in the leukocyte and lymphocyte concentrations of mice treated with 10, 20 and 40 g/kg Yanshu, but treatment with dexamethasone resulted in a significant decrease in the number of lymphocytes (P<0.01; Fig. 3B).

### Effect of Yanshu on the phagocytic index of monocyte and macrophages in mice

**Figure 4:**
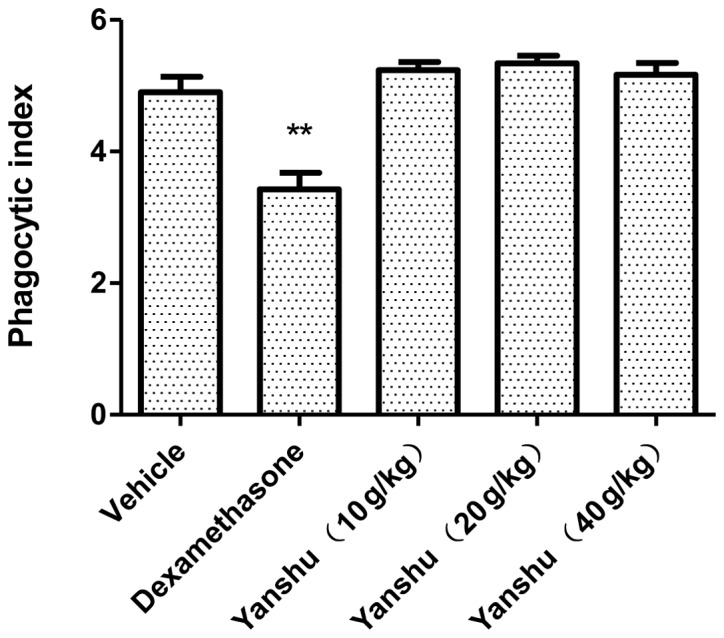
Effect of Yanshu on carbon particle clearance in mice. Results are expressed as the mean ± SD (n=10). ^**^P<0.01, vs. vehicle group.

The phagocytic index in Yanshu-treated mice did not change significantly compared with the vehicle group. However, dexamethasone-treated mice exhibited a significant decrease in the phagocytic index (P<0.01; Fig. 4).

## Discussion

The present study investigated the anti-inflammatory and antibacterial effects of Yanshu in relieving chronic pharyngitis, as well as the effect Yanshu has on the immune system. The results demonstrated that Yanshu inhibits chronic inflammation in animals and demonstrates antibacterial effects *in vitro*. As an anti-inflammatory drug, Yanshu does not affect immune function as compared with dexamethasone.

Chronic pharyngitis is chronic inflammation of the pharyngeal mucous membrane. In the present study, an ammonia-induced rabbit chronic pharyngitis model was established (10,11) to investigate the anti-inflammatory effects of Yanshu. The results revealed that Yanshu suppresses ammonia-induced chronic inflammation in pharyngeal tissue. Similar results were also observed in the cotton pellet-induced granulomatous inflammation model (12). The results indicate that Yanshu exhibits inhibitory effects against chronic inflammation.

Bacterial infection, particularly β-hemolytic *Streptococcus* infection, is one of the etiologies of pharyngitis (13,14). To investigate the antibacterial effects of Yanshu, the cylinder-plate method was used. This is a sensitive and popular method for antibacterial experiments and by measuring the diameter of the inhibition zones, the antibacterial effects of Yanshu were analyzed. Treatment with Yanshu significantly inhibited the growth of β-hemolytic *Streptococcus* and *Staphylococcus aureus*. Therefore, the results indicate that Yanshu exerts good antibacterial effects.

Pharyngitis is often a sign of immune dysfunction, as the immune system is usually robust enough to prevent throat infections. Chronic pharyngitis may be caused by immune dysfunction, as the immune system is insufficient in suppressing persistent infections, including Staphylococcal and Streptococcal infections. Therefore, treatment for pharyngitis needs to enhance immune function or at least not suppress function, unlike glucocorticoids that are commonly administered for pharyngitis (15–17). In the present study, Yanshu was found to have no inhibitory effects on immune function when compared with dexamethasone.

In conclusion, the present study demonstrated that Yanshu exhibits anti-inflammatory and antibacterial effects *in vivo.* In addition, Yanshu did not exhibit significant effects on the immune system *in vitro.* These observations support the use of Yanshu as a safe therapeutic agent for the treatment of chronic pharyngitis.

## Figures and Tables

**Table I:** Antibacterial activity of Yanshu *in vitro* (cylinder-plate method).

	Diameter of inhabiting circles (mm)	
	
Drug	*Staphylococcus aureus*	β-hemolytic *Streptococcus*
Saline	-	-
Penicillin 10 μg	44.3±1.6	19.3±0.6
Yanshu 2 g/ml	10.7±0.6	7.5±0.5
Yanshu 1 g/ml	8.7±0.6	6.7±0.6
Yanshu 0.5 g/ml	7.7±1.2	-
Yanshu 0.25 g/ml	8.7±0.6	-
Yanshu 0.125 g/ml	8.3±0.6	-
Yanshu 0.0625 g/ml	-	-
Yanshu 0.03125 g/ml	-	-

Values are the mean of triplicate experiments and ‘−’ indicates no activity.
